# Three-Dimensional Reconstruction of Fragment Shape and Motion in Impact Scenarios

**DOI:** 10.3390/s25185842

**Published:** 2025-09-18

**Authors:** Milad Davoudkhani, Hans-Gerd Maas

**Affiliations:** Institute of Photogrammetry and Remote Sensing, TUD Dresden University of Technology, 01069 Dresden, Germany; hans-gerd.maas@tu-dresden.de

**Keywords:** 3D shape reconstruction, high-speed camera, fragmentation, close range photogrammetry

## Abstract

Photogrammetry-based 3D reconstruction of the shape of fast-moving objects from image sequences presents a complex yet increasingly important challenge. The 3D reconstruction of a large number of fast-moving objects may, for instance, be of high importance in the study of dynamic phenomena such as impact experiments and explosions. In this context, analyzing the 3D shape, size, and motion trajectory of the resulting fragments provides valuable insights into the underlying physical processes, including energy dissipation and material failure. High-speed cameras are typically employed to capture the motion of the resulting fragments. The high cost, the complexity of synchronizing multiple units, and lab conditions often limit the number of high-speed cameras that can be practically deployed in experimental setups. In some cases, only a single high-speed camera will be available or can be used. Challenges such as overlapping fragments, shadows, and dust often complicate tracking and degrade reconstruction quality. These challenges highlight the need for advanced 3D reconstruction techniques capable of handling incomplete, noisy, and occluded data to enable accurate analysis under such extreme conditions. In this paper, we use a combination of photogrammetry, computer vision, and artificial intelligence techniques in order to improve feature detection of moving objects and to enable more robust trajectory and 3D shape reconstruction in complex, real-world scenarios. The focus of this paper is on achieving accurate 3D shape estimation and motion tracking of dynamic objects generated by impact loading using stereo- or monoscopic high-speed cameras. Depending on the object’s rotational behavior and the number of available cameras, two methods are presented, both enabling the successful 3D reconstruction of fragment shapes and motion.

## 1. Introduction

**Figure 1:**
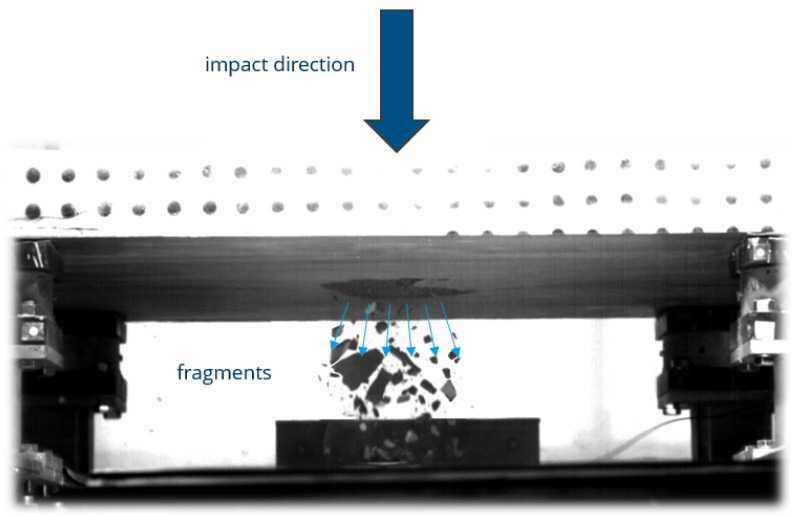
Fragmentation at the lower side of the slab during a drop tower impact test.

Accurate shape detection and 3D reconstruction of objects are essential in numerous scientific and engineering disciplines, as they enable the determination of key physical properties such as volume and mass. These properties are fundamental for modeling behavior, assessing performance, and understanding the physical characteristics. A wide variety of techniques has been developed to measure 3D shapes, each offering specific advantages and limitations depending on the object’s characteristics and environmental conditions. While many of these methods perform well in static or controlled scenarios, their effectiveness decreases significantly in dynamic situations involving high-speed motion or fragmentation. Three-dimensional reconstruction of objects in dynamic scenarios is also essential for analyses in dynamic phenomena such as impact experiments and explosions, where fragmentation occurs when a substantial amount of energy is delivered to a solid within a very short period of time. To characterize the material and structural response under impact loading, various experimental setups are employed. For reinforced concrete structures, split Hopkinson pressure and tension bars are commonly used for small-scale testing, while drop towers are utilized for large-scale impact experiments [1]. The number, shape, mass and velocity of the resulting fragments are crucial for understanding a wide range of phenomena, many of which have important practical implications. Determining these parameters in impact experiments is challenged by several factors, including limitations in the number of high-speed cameras due to constraints such as cost limitations, synchronization requirements, or spatial observation conditions, as well as fragment overlap, dust, and shadowing. These factors complicate accurate shape estimation and highlight the need for a suitable system setup and a robust method capable of handling such demanding scenarios. One particularly challenging case is impact testing in a drop tower, where a slab is impacted and fragmentation occurs predominantly on the lower side (Figure 1). In this region, the likelihood of occlusion due to dust, shadows, and overlapping fragments is significantly higher than in other setups. This makes reliable shape reconstruction particularly difficult and requires a tailored reconstruction method along with an optimized camera setup to address the specific constraints of these realistic and complex experimental conditions.

### 1.1. Close-Range Photogrammetric Object Surface Reconstruction Techniques

Photogrammetric methods provide powerful tools for precise shape determination. Methods such as optical imaging [2,3] and laser scanning [4,5] have been extensively used to estimate object geometry. However, these approaches are mostly designed for static objects, and their accuracy degrades significantly when applied to moving objects, as motion can cause distortions or hinder reliable data acquisition. Structured light techniques such as hierarchical phase shift methods [6,7] and coded light approaches [8,9] are widely used for high-precision 3D geometry acquisition in close-range applications [10]. These methods operate by projecting a sequence of structured light patterns onto the object’s surface, typically requiring the capture of 8 to 12 successive images, each with a different phase or code. As a result, these methods require multiple images to be taken in rapid succession, each with a different projected pattern. If the object moves during this sequence, the captured patterns become inconsistent, leading to distorted measurements and reconstruction errors [11]. This makes structured light techniques inherently unsuitable for capturing moving objects. Also, approaches that combine the benefits of phase shift and coded light to maximize measurement efficiency [12] do not overcome this fundamental limitation. In contrast, stereo photogrammetric methods based on multiple synchronized cameras identify and match natural texture features across images captured simultaneously from different viewpoints, allowing robust 3D reconstruction even in dynamic scenes.

### 1.2. Related Work

A recent experimental method for studying fragmentation caused by hypervelocity impacts presented by [13] uses a thin laser sheet to illuminate a debris cloud, which is recorded using a high-speed camera. The resulting image sequence is analyzed to obtain spatio-temporal data for individual fragments, including their 2D geometry and velocity. To extract this information, fragment contours are identified by applying an optimized threshold that separates each particle from the background. In addition to experimental approaches, computational methods have also been developed for the characterization of debris clouds an essential aspect of understanding the physical behavior of fragmenting structures. One such method, introduced by [14], uses convex hull construction and iterative expansion to identify fragment boundaries and extract key parameters from large-scale 3D point cloud data. This enables a detailed analysis of fragment size, mass, velocity, and momentum distributions. To understand the velocity distribution of fragments resulting from hypervelocity impacts, an experimental setup and analysis method were developed to enable the tracking and measurement of individual fragment velocities. The system employs two synchronized high-speed cameras with split images, providing four distinct views to accurately capture fragment motion [15]. Mirror systems may depict an interesting option to obtain two or even four stereoscopic views from a single camera, sacrificing spatial resolution for stereo [16]. Two other recent studies on fragmentation include the work by [17], in which rockfall events are recorded using a high-speed camera system, and the trajectories of falling unbroken blocks and the fragmentation of the blocks upon impact are analyzed to extract parameters such as velocity and acceleration. This analysis allow the researchers to estimate the impact energy, the dissipated energy, and the resulting fragmentation patterns. In another study [18], the fragmentation of microparticles resulting from high-speed impacts is systematically analyzed using an image-based tracking method. High-speed imaging is employed to capture the extremely rapid events of particle impact, and the resulting fragments are subsequently tracked and quantified.

Such experimental investigations are highly relevant when considering space-related fragmentation events, such as on-orbit explosions of rocket components or secondary breakups caused by mutual fragment collisions, which represent a major source of untrackable space debris [19]. For safety and mitigation purposes, it is, therefore, crucial to analyse the resulting fragments. The orbits of fragments are determined by their velocity; however, the lifetime of the orbits is limited by the atmospheric drag, which mainly depends on the shape of the fragments. The probability of impact of debris pieces with a space craft and the resulting damage can be calculated from their velocity, mass, size, and shape [20]. However, acquiring sharp and accurate images of such high-speed fragments presents significant challenges. Motion blur is a common issue in image acquisition, typically caused by rapid object movement, camera shake, or long exposure times. This is especially problematic when capturing fast-moving objects that travel a distance greater than their own size during a single exposure. Reconstructing the shape and motion of such objects is highly challenging. Several studies have addressed this problem by recovering the textured 3D shape and motion parameters from single blurred images, and by extending these approaches to video sequences to jointly optimize shape, texture, and motion across frames. The methods simulate motion-blurred images of the reconstructed objects and optimize their shape, texture, and trajectory by minimizing the difference between the simulated and observed images [21,22]. However, motion blur can be effectively avoided when using high-speed cameras with appropriate exposure times and frame rates and is not a concern in this study, as high-speed cameras with appropriate exposure times and frame rates are employed.

### 1.3. Goals and Structure

The paper focuses on accurate shape detection and 3D reconstruction of fragments in impact experiments captured by high-speed cameras, using suitable system configurations based on one or two high-speed cameras and a processing chain consisting of photogrammetric, computer vision, and artificial intelligence (AI) techniques. The paper is structured as follows: in Section 2, the experimental setup is described. Section 3 and Section 4 present the methodology for 3D reconstruction, where object motion is categorized into two types and the corresponding analysis methods for each category are described in detail. Section 5 presents the results and validation, followed by a discussion of the findings in Section 6. Finally, Section 7 concludes the paper.

## 2. Experimental Setup

**Figure 2:**
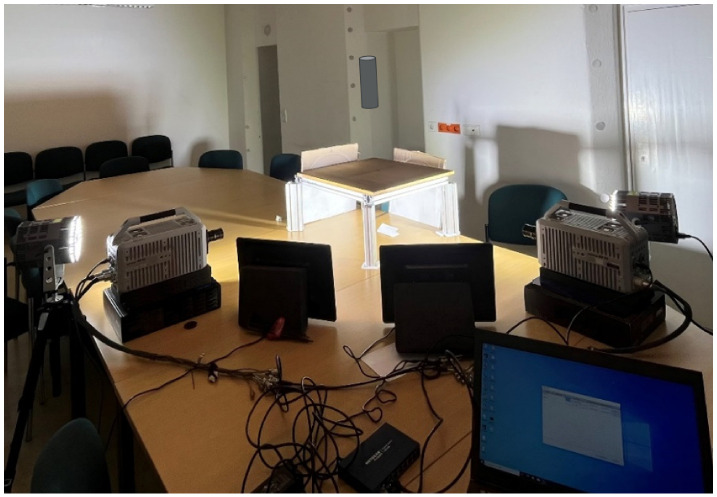
Experimental setup (90-degree configuration of two Photron cameras).

A 90-degree configuration of two synchronized high-speed cameras to obtain dual views for shape estimation of moving objects was employed. As an imaging device, two high-speed cameras Photron Fastcam SA X2, (Photron Ltd., Tokyo, Japan) at full resolution (1024 × 1024 px at 12,500 fps), were used. The main technological specifications of this camera are listed in Table 1. A 1kW-LED-4438 lighting system with 100,000 lm in continuous operation was used. Figure 2 shows the experimental setup.

## 3. Methodology

**Figure 3:**
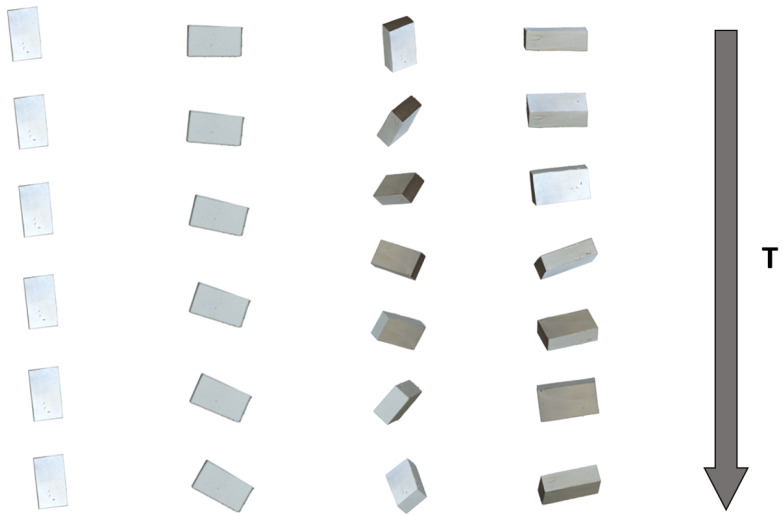
Rotational and non-rotational falling scenarios of simulated fragments: objects exhibiting significant rotation, and objects remaining stable or with negligible rotation. T represents the translational motion of the object over time.

In this paper, we focus on the 3D reconstruction of fragments generated in complex impact experiments, including impact testing performed in a drop tower. As presented in Figure 3, a moving object may rotate significantly as it moves, or it may remain non-rotating or be only slightly rotating. For determining the shape of a moving object, it is essential to know this to select the proper method. Obviously, significant object rotation depicts a pre-requisite for the application of monoscopic image sequence-based 3D reconstruction approaches.

In the first part, our focus is on objects without significant rotation and without any prior information about the object’s shape, which is particularly important in impact experiments where fragmentation occurs and the shape of the resulting fragments is not known in advance. For this scenario, we employ a 90-degree setup of two synchronized high-speed cameras to achieve two views for shape estimation without any pre information about objects, which do not or only slightly rotate during the movement. The 90-degree configuration of two synchronized high-speed cameras improves the chance of capturing the view of the rear or occluded region of the object, which is typically not visible in standard stereo setups with shorter baselines. This extended spatial coverage significantly enhances the completeness of the reconstructed 3D geometry. The captured image sequences of the in-flight fragments are analyzed by identifying the fragments in each view using a threshold-based background subtraction algorithm in shadow- and overlap-free conditions, or the trained Mask R-CNN model in case of overlapping and shadowed scenarios. The corresponding fragments are matched across views using epipolar geometry, their contours are extracted and finally the triangulation of their 3D positions in every frame is performed using spatial intersection. The shape of the fragment is determined based on its contours extracted in the two different projections. This results in a method capable of 3D tracking and 3D model reconstruction of objects during the motion.

The second approach (see Section 4.2) shifts attention to the analysis of rotating objects. These objects are reconstructed in 3D during motion using a Structure-from-Motion (SfM) approach [24,25] on the basis of monoscopic image sequences, wherein the object rotation is translated into virtual camera motion.

## 4. Description and Experimental Evaluation of the Proposed Methods

This section presents the processing chain of the proposed methods under different motion scenarios. The analysis is structured into two parts: Section 4.1 focuses on cases involving non- or slowly rotating motion. While Section 4.1.1 discusses objects under non-overlapping and non-shadowed conditions, Section 4.1.2 introduces an AI-based method for complex scenarios involving overlapping objects and shadows. Section 4.2 addresses the challenges of reconstructing objects undergoing significant rotational motion from monoscopic image sequences.

For each case, qualitative and quantitative results are provided in Section 5 to assess the performance and robustness of the proposed techniques.

Dynamic fragmentation experiments were conducted under laboratory conditions, in which stone samples were subjected to impact and allowed to fall down in order to simulate fragmentation occurring on the underside of slabs during drop tower impact scenarios.

### 4.1. Non-Rotating or Low-Rotation Motion

#### 4.1.1. Image Analysis and 3D Shape Reconstruction

An initial crucial step involved calibrating the high-speed camera. A self calibration approach [7] was employed to determine the camera parameters of the experimental setup. An image block consisting of 36 images of a calibration field was captured in a camera calibration scheme, as suggested in [26]. The resulting standard deviation of the unit weight was $\sigma_{0}$ = 0.48 µm (0.024 pixel) for the left camera and $\sigma_{0}$ = 0.46 µm (0.023 pixel) for the right camera. The average standard deviation of object point coordinates obtained from bundle block adjustment was for the left camera ${\mathrm{RMS}}_{X}$ = 2 µm, ${\mathrm{RMS}}_{Y}$ = 2 µm and ${\mathrm{RMS}}_{Z}$ = 3 µm and for second one ${\mathrm{RMS}}_{X}$ = 2 µm, ${\mathrm{RMS}}_{Y}$ = 2 µm and ${\mathrm{RMS}}_{Z}$ = 2 µm, where ${\mathrm{RMS}}_{X}$, ${\mathrm{RMS}}_{Y}$, and ${\mathrm{RMS}}_{Z}$ represent the root mean square errors of the object points along the X, Y, and Z directions, respectively. The coordinate system was oriented with the Z-axis in the camera viewing direction. The calibration parameters according to Brown [27] were used to correct the images for distortions.

Segmentation of the fragment stereo image sequences with the goal of object identification in each image frame was performed by threshold-based background subtraction, a method for foreground detection in image sequences, where each pixel of the current frame is compared to a background model, and pixels with differences above a predefined threshold are classified as foreground objects [28]. This operation was applied to every frame in the two image sequences, yielding a set of detected object candidates with associated 2D centroid coordinates and timestamps. This method is effective; however, for object detection in scenarios involving overlap, shadows, and dust, a more robust and appropriate alternative method is necessary. A solution to these challenges is presented in Section 4.1.2. Identifying corresponding features in images captured simultaneously from two synchronized cameras enables 3D point reconstruction through spatial intersection, making correspondence a key step for determining fragment positions and velocities. To facilitate this process, epipolar geometry was employed by computing epipolar lines and epipoles. The computation of these geometric entities relies on two key matrices: the Fundamental Matrix (F) and the Essential Matrix (E). The Essential Matrix encodes the relative rotation and translation between the two camera views, thereby capturing the position and orientation of the second camera with respect to the first. The Fundamental Matrix contains the same geometric information as the Essential Matrix, but additionally incorporates the intrinsic parameters of both cameras [29]. This allows us to relate corresponding points between the two views directly in pixel coordinates. To estimate the Fundamental Matrix using the eight-point algorithm from matching points from both the images, a minimum of eight point correspondences is required. However, using more than eight points is generally preferred to improve accuracy. Twelve distributed steel spheres with a diameter of 13.09 mm were used for estimation of the matrices and scaling. The calculation of these matrices was performed using 12 point correspondences, with RANSAC employed to handle outliers and achieve a robust estimation. To establish object correspondences, the epipolar line for each detected fragment’s center point in the left camera frames was computed in the corresponding right camera frames. Matching points were then identified by minimizing the perpendicular distance of candidate points to the respective epipolar lines. The object in View 1 and its corresponding object in View 2 for each frame were marked and stored together as a correspondence in the database. Once the correspondences were determined, spatial intersection was performed to determine the 3D position of the objects. To track these objects over time, prediction was performed using a Kalman filter. Detections across consecutive frames were associated using the Hungarian algorithm [30], which computes the minimum-cost matching based on a cost matrix defined by the Euclidean distance between predicted and measured 3D positions. Using the obtained 3D trajectories and the corresponding timestamps, the motion velocities of the fragment swarm can be calculated. For object shape reconstruction, the contours of the matched objects in View 1 and View 2 were extracted, extruded and intersected in object space to generate a 3D representation. After reconstructing the 3D model of the object, the volume of the generated closed 3D mesh was calculated. If the physical density of the object material is known, the total mass can be estimated by multiplying the calculated volume.

In this procedure, it is assumed that the scenes involve non-overlapping objects and lack visual disturbances such as shadows or dust. Under such clean conditions, conventional techniques for detection and segmentation prove sufficient.

#### 4.1.2. AI-Based Object Detection and Segmentation in Overlapping and Shadowed Scenarios

One of the main challenges in multi-object 3D reconstruction and tracking lies in the accurate detection and segmentation of individual objects, particularly under complex conditions such as shadows, dust, and overlapping objects. These challenges are commonly encountered in dynamic experiments involving impacts or explosions, where traditional image processing methods, such as thresholding or background subtraction, often fail due to their sensitivity to lighting variations and their lack of robustness in cluttered or rapidly changing scenes. In this context, advanced AI models like Mask R-CNN offer an effective solution. The use of Mask R-CNN as a deep learning framework designed for object instance segmentation provides a significant advantage in such scenarios, as it enables precise object detection, contour extraction, and segmentation. This facilitates more accurate object tracking and contributes to a higher-fidelity 3D model reconstruction of multiple objects in motion. In addition to generating bounding boxes for object detection, Mask R-CNN also produces segmentation masks that delineate the exact pixels belonging to each detected instance [31]. This capability makes Mask R-CNN particularly well-suited for accurately classifying and localizing objects during motion, as it enables pixel-level tracking and analysis of object contours throughout the sequence.

### 4.2. Rotating Motion

In explosion and impact experiments, fragments frequently exhibit rotational motion. This part is dedicated to the detailed analysis of such rotating bodies. Obviously, the procedure, as described in Section 4.1.1, can also be applied in the case of rotating fragments. However, significant object rotation allows the use of a monocular approach, meaning that only a single camera is needed for reconstruction and segmentation. In this scenario, Structure-from-Motion (SfM) is used for 3D model reconstruction of an object rotating in front of a fixed camera. When the object rotates, the resulting images have different perspectives, which is mathematically equivalent to when the camera would move around a stationary object. The SfM algorithm estimates the relative orientations of the object in this case and reconstructs its 3D geometry based on this, provided that enough visual features can be identified and matched across multiple views. This technique enables 3D shape reconstruction of rotating objects from image sequences acquired by a single high-speed camera. For 3D object tracking using only a single camera, the presented photogrammetric approach based on inverse spatial resection for 3D object tracking can be employed [32]. The method enables the direct computation of velocity, acceleration, and rotational parameters of a moving object in 3D space, allowing the determination of the object’s position and trajectory from image sequences captured by a single high-speed camera. The combination of these two approaches for 3D tracking and 3D shape reconstruction using only a single camera also offers potential for a wide range of other applications.

If a rotating object is recorded using two high-speed cameras from different viewing angles, e.g., 90 degrees apart, the image sequences from both cameras can be used for 3D reconstruction of each object using the SfM approach. Either a single 3D model can be reconstructed from the combined image data of both cameras, or two separate models can be reconstructed independently from each camera’s images and subsequently merged to obtain a more complete 3D representation of the object.

## 5. Results

This section presents the results of the proposed methods for 3D reconstruction of moving objects. Section 5.1 presents results obtained with a stereo high-speed camera under shadow-, and overlap-free conditions, as well as in overlapping and shadowed scenario for objects with non-rotating or low-rotation motion, while Section 5.2 presents results obtained by a single camera system for an object that was rotating during its fall.

### 5.1. Non-Rotating or Low-Rotation Motion

#### 5.1.1. A 3D Reconstruction Under Dust-, Shadow-, and Overlap-Free Conditions

**Figure 4:**
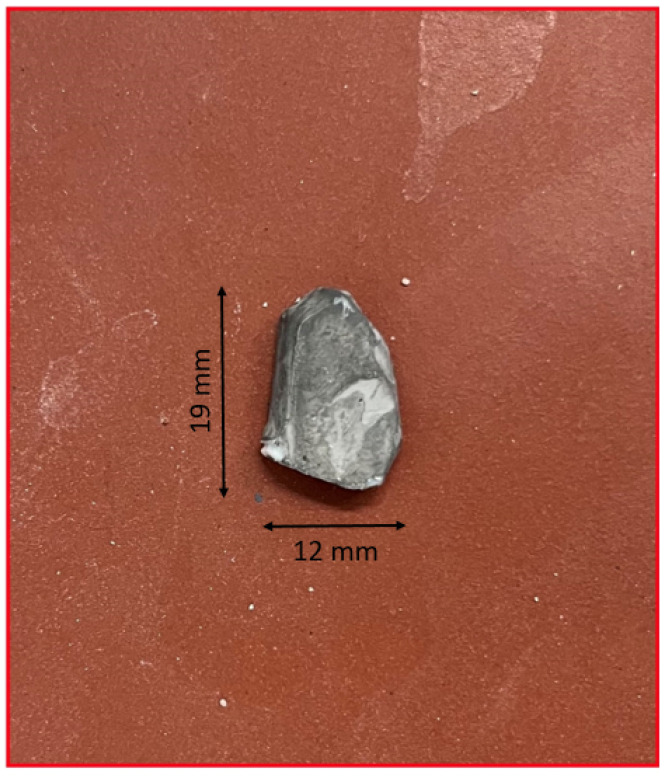
Image of the object.

**Figure 5:**
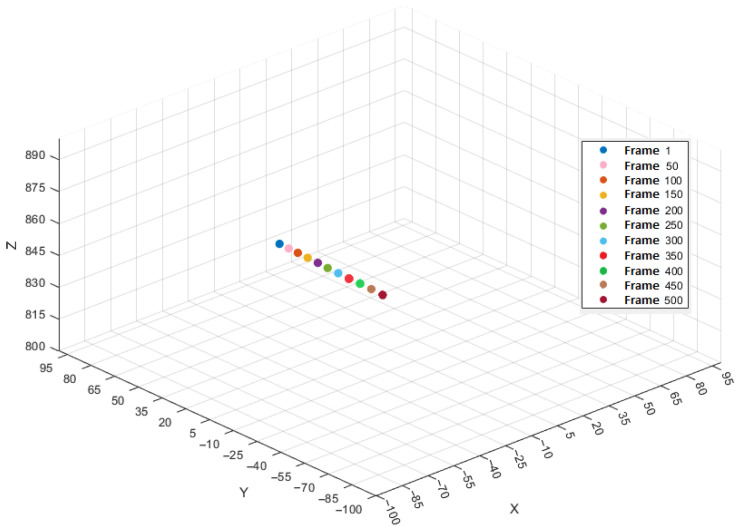
Trajectory of the object over each 50th frame.

**Figure 6:**
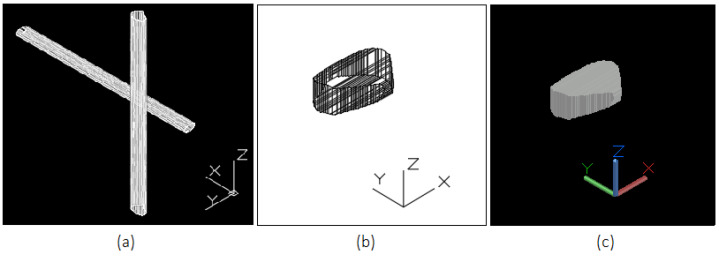
(**a**) Extrusion of extracted contours from View 1 and 2 for 3D reconstruction of the fragment, (**b**) reconstructed 3D wireframe model of a falling fragment without notable rotation during motion obtained from the intersection of extruded contours, (**c**) the same reconstructed 3D model in solid view.

The results of the tracking and 3D reconstruction of a single falling fragment, shown in Figure 4, without significant rotation are shown in Figure 5 and Figure 6. In total, 500 frames were recorded for analysing the object motion and the recording duration was 40 ms (time difference between frames = 80 µs). Object detection and segmentation for the proposed contour-based 3D reconstruction method were performed using thresholding and background subtraction due to the absence of overlapping objects, shadows, or dust in the scene, allowing for a clean separation of foreground elements.

The average velocity between frames 1 and 100 with a time difference of 0.008 s per time step is 29.5 mm/s in the X-direction, 1496.6 mm/s in the Y-direction, and 21.8 mm/s in the Z-direction. The resulting 3D velocity of the object during this interval is 1497.1 mm/s, dominated by the motion in the Y-direction. The object’s trajectory, sampled at intervals of 50 frames, is illustrated in Figure 5. Note that the coordinate system was oriented with the Z-axis in the camera viewing direction and the motion is in the Y direction.

**Figure 7:**
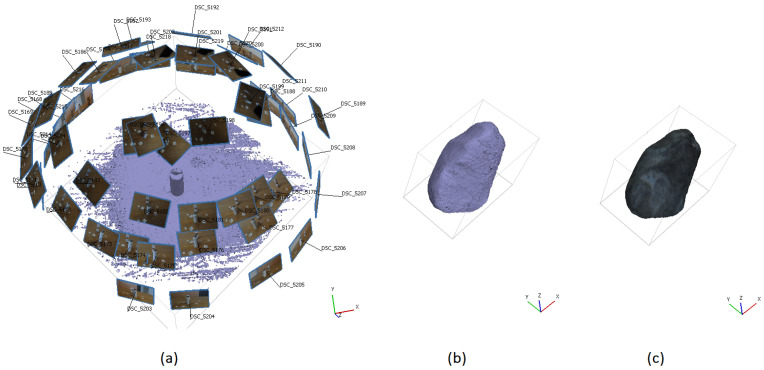
(**a**) The configuration of camera poses, as well as the generated dense point cloud of the fragment in a static position after the experiment obtaind from SfM for validation: (**b**) 3D point cloud representation of the fragment, (**c**) reconstructed 3D mesh.

To validate the results, the volume of the 3D model reconstructed using the proposed method and configuration for the moving object ($655.1\;{\mathrm{mm}}^{3}$) is compared to the 3D model of the same object in a static state of $649.2\;{\mathrm{mm}}^{3}$ obtained by an SfM approach after the experiment (Figure 7). The difference in volume between the two reconstructions is $5.9\;{\mathrm{mm}}^{3}$, corresponding to a deviation of 0.9%, which demonstrates a high degree of consistency and indicating that the proposed method provides good volumetric estimation despite object motion. The tracked object was weighed in the laboratory and has a mass of 1.8 g. Multiplying the volume by the physical density of the material, we obtain a mass of 1.816 g. This indicates a mass difference of 0.016 g between the real and reconstructed object, which fits well to the weighed mass of 1.8 g.

#### 5.1.2. Experimental Results and Performance Evaluation of Mask R-CNN in Overlapping and Shadowed Scenarios

**Figure 8:**
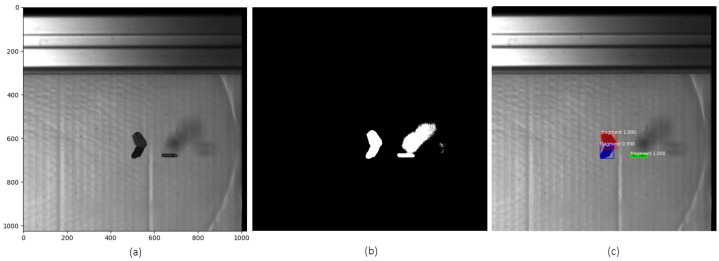
Comparison of object detection methods. (**a**) Original image, (**b**) detection results using thresholding and background subtraction, (**c**) Mask R-CNN detection results.

In the following, we evaluate the performance of the Mask R-CNN model in scenarios involving multiple overlapping and shadowed fragments. A training dataset was created consisting of 100 images randomly selected from recorded image sequences of 26 different experiments involving falling objects with varying quantities, sizes, and directions after impact. The selected images were subsequently annotated with mask labels to enable accurate instance segmentation. Data augmentation refers to techniques that artificially expand the size of a training dataset in order to reduce overfitting, eliminating the need for additional data collection [33,34]. To enhance the model’s ability to detect and segment objects in motion, data augmentations were employed on the training dataset. These included random rotation, brightness adjustment, sharpness and contrast modification, as well as horizontal and vertical flipping. A total of 300 images were created and added to the training dataset. The model was trained on this dataset. Similarly, a validation dataset consisting of 43 images was created using a separate set of images randomly selected from the another 10 test sequences, which were also annotated with corresponding mask labels. The trained Mask R-CNN model reached a mean Average Precision (mAP) of 0.928 on the training dataset, indicating that the model successfully learned to detect and segment instances with high accuracy. Additionally, the qualitative results, including visual comparison to traditional methods, confirm the model’s effectiveness in performing reliable instance segmentation. To further evaluate the model’s performance in complex scenarios, an experiment was conducted focusing on overlapping objects. In this experiment, two of the three falling objects appeared consistently overlapping throughout the entire trajectory in the views of both cameras. Additionally, shadows cast by the objects were visible in the captured images. A total of 500 frames of the falling objects were recorded by the two cameras for analysing the object motion. Figure 8 shows some detection results obtained using thresholding and background subtraction, as well as Mask R-CNN. While the traditional method failed to correctly detect overlapping objects and mistakenly includes shadows, Mask R-CNN accurately detects and segments overlapping objects and properly disregards shadows, avoiding their misclassification as separate objects. Visual inspection of the predicted masks further confirms the robustness of the Mask R-CNN model, demonstrating precise instance segmentation even under challenging conditions such as object overlap and varying illumination.

**Figure 9:**
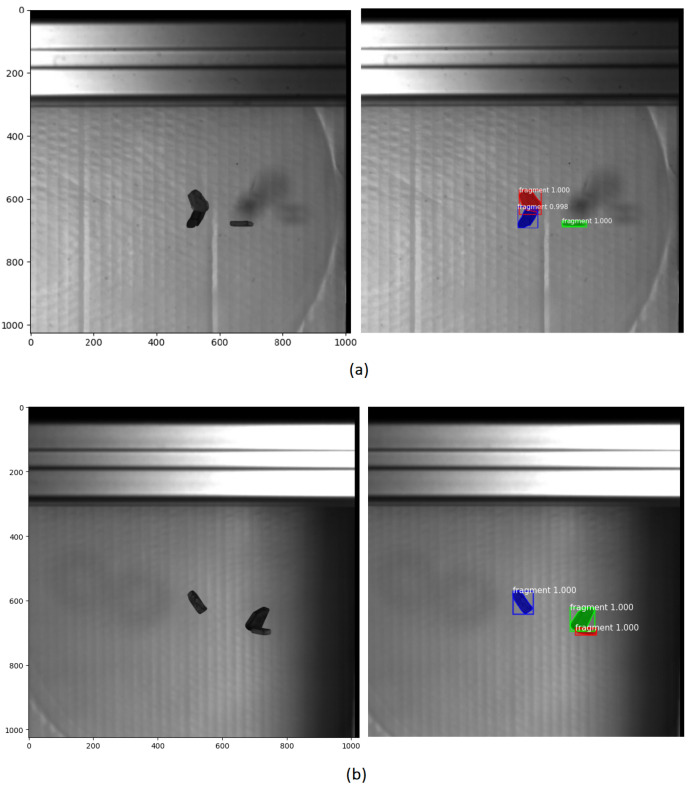
Detection and segmentation results using Mask R-CNN for frame 270 from (**a**) left and (**b**) right cameras.

**Figure 10:**
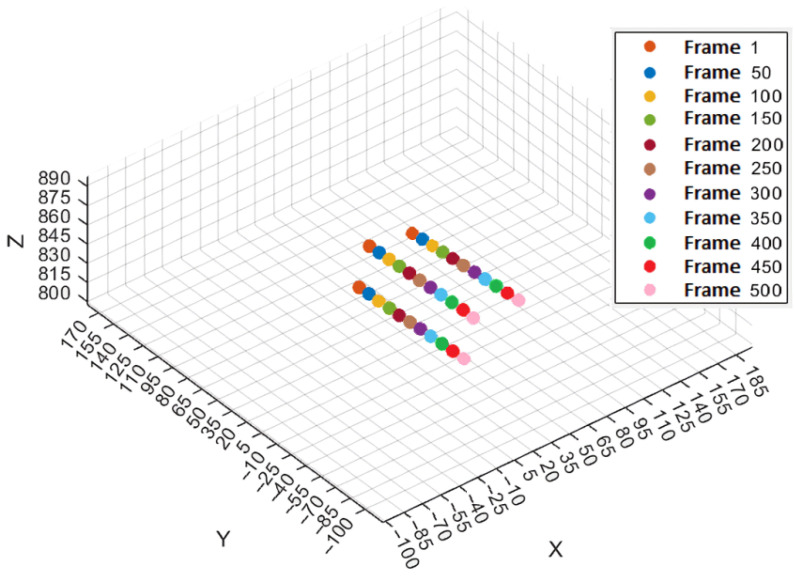
Trajectories of the three objects over each 50th frame.

**Figure 11:**
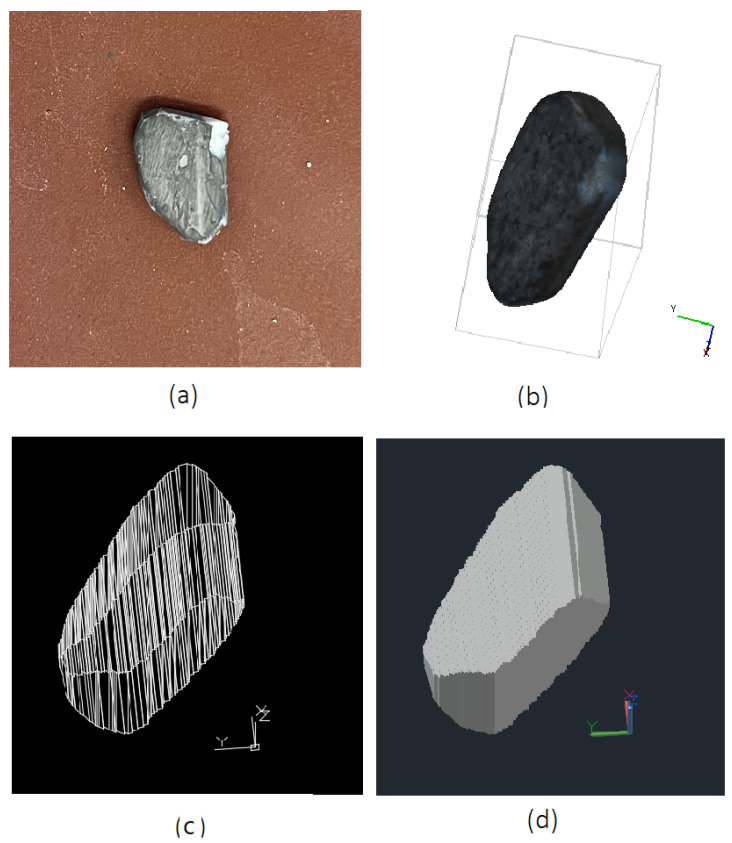
(**a**) Photo of the object, (**b**) Three-dimensional reference mesh of the fragment in a static position after the experiment using SfM, (**c**,**d**) Reconstructed 3D model of an overlapping falling fragment without notable rotation during motion using the proposed method, shown in wireframe and solid views.

Figure 9 and Figure 10 show the detection and segmentation results produced by Mask R-CNN for a captured frame from both the left and right cameras, as well as the motion trajectory of all three detected and segmented objects sampled every 50 frames. Additionally, the reconstructed 3D model of one of the overlapping objects during motion and the generated static mesh of the object obtained via Structure-from-Motion (SfM) after the experiment are presented in Figure 11.

The average velocity of this object between frames 50 and 150 with time difference of 0.008 s per step is 71.6 mm/s in the X-direction, 2454.4 mm/s in the Y-direction, and 57.8 mm/s in the Z-direction. The resulting average 3D velocity of the object during this interval is 2456.1 mm/s.

For validation purposes, the volume of the reconstructed 3D model for the moving object ($664.1\;{\mathrm{mm}}^{3}$) was compared with that of the same object captured in a static condition using Structure-from-Motion approach ($649.2\;{\mathrm{mm}}^{3}$). The comparison revealed a volume deviation of $14.9\;{\mathrm{mm}}^{3}$ between the two reconstructions (corresponding to 2.3%). The results for mass calculation indicate that the reconstructed model exhibits a mass difference of 0.041 g to the reference mass (1.8 g).

### 5.2. Rotating Motion

**Figure 12:**
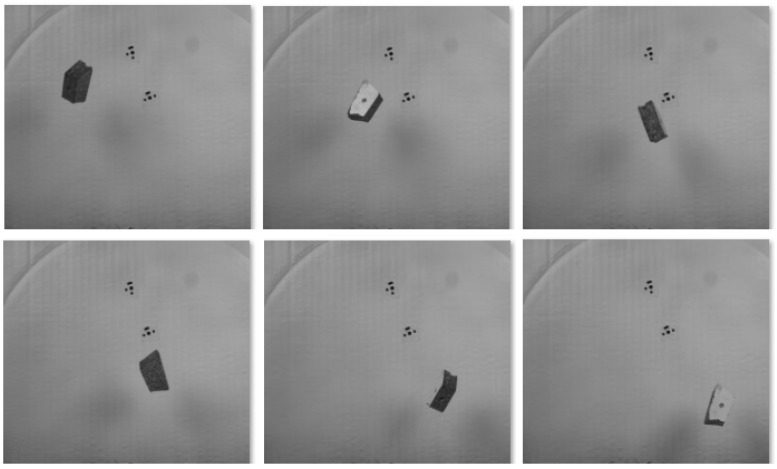
Visualization of the object’s rotational motion using six selected frames from a 200-frame sequence.

**Figure 13:**
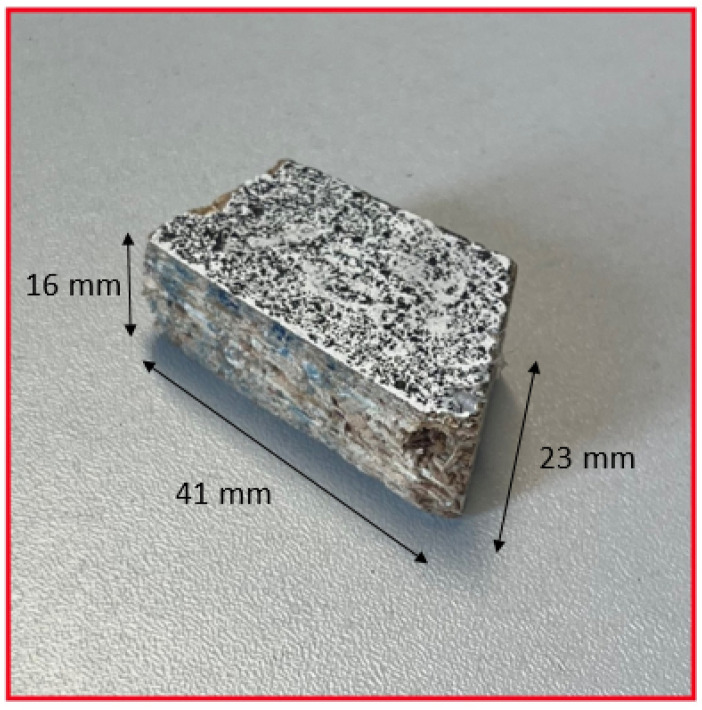
Photo of the object.

**Figure 14:**
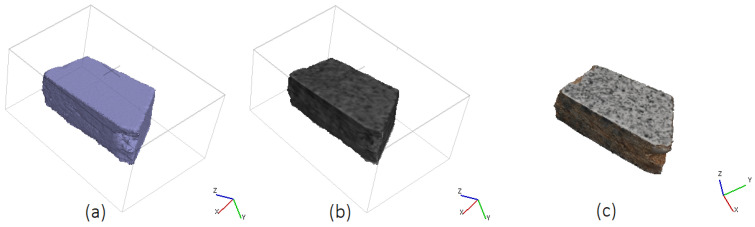
Three-dimensional reconstruction of the fragment using SfM during motion: (**a**) point cloud representation, (**b**) reconstructed mesh, (**c**) three-dimensional reference mesh of the fragment in a static position.

In the following experiment, a wooden object was recorded while falling and rotating using single camera (Figure 12). The object and its 3D reconstruction using the methods, as described in Section 4.2, are shown in Figure 13 and Figure 14.

The generated point cloud consisted of 427,995 points. Subsequently, a mesh was created from the point cloud, comprising about 210,958 vertices and 105,905 faces, which allowed for an accurate volumetric analysis.

The volume of the 3D model reconstructed using SfM during the motion of the moving object (11,414 ${\mathrm{mm}}^{3}$) was compared to the reconstructed 3D model of the same object in a static state using SfM with 20 images (11,176 ${\mathrm{mm}}^{3}$). The comparison revealed a volumetric discrepancy of ($238\;{\mathrm{mm}}^{3}$) between the two models, which corresponds to a deviation of 2.1%. The reconstructed model’s mass deviated by approximately 0.17 g from the actual mass of the object, which was weighed to be 8 g.

## 6. Discussion

Performing 3D reconstruction of objects in motion during dynamic events enables the accurate capture of transient geometrical changes and dynamic deformation processes that occur during the event. It allows for tracking and 3D model reconstruction of all fragments immediately after impact, including small and fast-moving ones that may be difficult or impossible to identify unambiguously after the event. If 3D fragment model reconstruction is only performed after an experiment, it is often unclear which reconstructed fragment corresponds to which object in the recorded images, complicating the reliable determination of fragment velocities and kinetic energies and thus limiting the ability to analyze energy dissipation. Additionally, fragments may undergo secondary deformations or even break further after impact, for instance, upon hitting the ground, leading to inaccurate or misleading 3D models if reconstructed only post hoc. The experimental results from the two proposed methods demonstrate the great potential of the methods in reconstructing 3D models of fast-moving objects of unknown geometry during motion. The first method (two-camera based) is particularly effective for reconstructing the shapes of small, textureless fragments, while the second method is better suited for large fragments and those with complex geometries. Mask R-CNN proved to be a solution to key challenges in 3D shape reconstruction, such as handling overlapping objects, shadows, and dust. It enables accurate detection and segmentation of moving objects under visually complex conditions.

The methods are well-suited for impact experiments in material testing, especially in drop tower tests where concrete slabs are subjected to impact loading. Tracking and accurate 3D reconstruction of the resulting fragments during impact experiments is critical for understanding dynamic behavior, and doing important analysis like energy dessipation. While high-speed cameras with a framerate of 12,500 fps turned out to be fast enough for the experiments at hand, the methods can also be applied to experiments with much higher speeds, employing ultra-high-speed cameras with framerates of up to 7 Mfps [35]. The presented methods are scalable over a wide range of object sizes and can be applied to different object sizes by only adapting the camera distance and the camera network configuration.

## 7. Conclusions

Three-dimensional model reconstruction and tracking of moving objects deliver essential information for understanding their geometric structure and dynamic behavior. This is particularly important for the analysis of fragments in impact experiments or flying debris generated during explosions. This paper demonstrates that, by combining photogrammetric, computer vision, and artificial intelligence techniques, it is possible to reconstruct more accurate 3D models of moving objects captured by high-speed cameras. Two methods for 3D shape reconstruction of fast-moving objects are presented in this study. By selecting the reconstruction method based on the object’s rotational behavior and the number of available cameras, shapes of a significant portion of fragments after impact or explosion, which vary widely in size and originate from different regions of the specimen, with no prior information about their shape, are successfully reconstructed. This paper and the methods presented herein will form part of a subsequent publication involving the analysis of data from real impact experiments.

## Figures and Tables

**Table 1:** Main specifications of the Photron Fastcam SA-X2 high-speed camera [23].

Sensor type	CMOS, monochrome model
Pixel Size	20 µm × 20 µm
Sensor Size	20.48 × 20.48 mm
Maximum Resolution	1024 × 1024 px at 12,500 fps
Fill Factor	58%
Minimum Exposure	Global electronic shutter to 1 µs

## Data Availability

Experimental raw data and results are available upon request from the author responsible for the correspondence.

## Abbreviations

The following abbreviations are used in this manuscript:

SfM	Structure from Motion
AI	Artificial Intelligence
F	Fundamental Matrix
E	Essential Matrix
${\mathrm{RMS}}_{X}$	Root Mean Square error along the X direction
${\mathrm{RMS}}_{Y}$	Root Mean Square error along the Y direction
${\mathrm{RMS}}_{Z}$	Root Mean Square error along the Z direction

## References

[1] Leicht L., Beckmann B., Curbach M. (2021). Influences on the structural response of beams in drop tower experiments. Civ. Eng. Des..

[2] Gallo A., Muzzupappa M., Bruno F. (2014). 3D reconstruction of small sized objects from a sequence of multi-focused images. J. Cult. Herit..

[3] Patrucco G., Giulio Tonolo F., Sammartano G., Spanò A. (2022). SFM-based 3D reconstruction of heritage assets using UAV thermal images. Int. Arch. Photogramm. Remote Sens. Spat. Inf. Sci..

[4] Engin I.C., Maerz N.H. (2019). Size distribution analysis of aggregates using LiDAR scan data and an alternate algorithm. Measurement.

[5] Arayici Y. (2007). An approach for real world data modelling with the 3D terrestrial laser scanner for built environment. Autom. Constr..

[6] Andresen K. (1986). Das Phasenshiftverfahren zur Moiré-Bildauswertung. Optik.

[7] Luhmann T., Fraser C., Maas H.G. (2015). Sensor modelling and camera calibration for close-range photogrammetry. ISPRS J. Photogramm. Remote Sens..

[8] Stahs T.G., Wahl F.M., Gruen A., Baltsavias E.P. (1990). Fast and robust range data acquisition in a low-cost environment. Proceedings of the Close-Range Photogrammetry Meets Machine Vision.

[9] Batlle J., Mouaddib E., Salvi J. (1998). Recent progress in coded structured light as a technique to solve the correspondence problem: A survey. Pattern Recognit..

[10] Bell T., Li B., Zhang S. (2016). Structured Light Techniques and Applications. Wiley Encyclopedia of Electrical and Electronics Engineering.

[11] Lu L., Xi J., Yu Y., Guo Q. (2013). New approach to improve the accuracy of 3-D shape measurement of moving object using phase shifting profilometry. Opt. Express.

[12] Malz R., Burkhardt H., Höhne K.H., Neumann B. (1989). Adaptive Light Encoding for 3-D-Sensing with Maximum Measurement Efficiency. Mustererkennung 1989, Proceedings of the 11. DAGM-Symposium Hamburg, Hamburg, Germany, 2–4 October 1989.

[13] Watson E., Gulde M., Hiermaier S. (2017). Fragment Tracking in Hypervelocity Impact Experiments. Procedia Eng..

[14] Liang S.C., Li Y., Chen H., Huang J., Liu S. (2013). Research on the technique of identifying debris and obtaining characteristic parameters of large-scale 3D point set. Int. J. Impact Eng..

[15] Watson E., Kunert N., Putzar R., Maas H.G., Hiermaier S. (2019). Four-View Split-Image Fragment Tracking in Hypervelocity Impact Experiments. Int. J. Impact Eng..

[16] Putze T., Raguse K., Maas H.G. (2007). Configuration of multi mirror systems for single high speed camera based 3D motion analysis. Proc. SPIE-Int. Soc. Opt. Eng..

[17] Prades-Valls A., Corominas J., Lantada N., Matas G., Núñez-Andrés M.A. (2022). Capturing rockfall kinematic and fragmentation parameters using high-speed camera system. Eng. Geol..

[18] Weindorf B.J., Morrison M., Lowe K.T., Ng W., Loebig J. (2025). Fragment tracking for microparticle breakage resulting from high-speed impacts. Powder Technol..

[19] Filho W.L., Abubakar I.R., Hunt J.D., Dinis M.A.P. (2025). Managing space debris: Risks, mitigation measures, and sustainability challenges. Sustain. Futur..

[20] Johnson N., Krisko P., Liou J.C., Anz-Meador P. (2001). NASA’s new breakup model of evolve 4.0. Adv. Space Res..

[21] Rozumnyi D., Oswald M.R., Ferrari V., Matas J., Pollefeys M. DeFMO: Deblurring and Shape Recovery of Fast Moving Objects. Proceedings of the 2021 IEEE/CVF Conference on Computer Vision and Pattern Recognition (CVPR).

[22] Rozumnyi D., Oswald M.R., Ferrari V., Pollefeys M. Motion-From-Blur: 3D Shape and Motion Estimation of Motion-Blurred Objects in Videos. Proceedings of the IEEE/CVF Conference on Computer Vision and Pattern Recognition (CVPR).

[23] Photron (2017). Photron Fastcam SA-X2 Datasheet (8 Juli 2025).

[24] Ullman S. (1979). The interpretation of structure from motion. Proc. R. Soc. Lond. Ser. B. Biol. Sci..

[25] Westoby M., Brasington J., Glasser N., Hambrey M., Reynolds J. (2012). ‘Structure-from-Motion’ photogrammetry: A low-cost, effective tool for geoscience applications. Geomorphology.

[26] Godding R., Beiser L., Lenz R.K. (1993). Photogrammetric method for the investigation and calibration of high-resolution camera systems. Proceedings of the Recording Systems: High-Resolution Cameras and Recording Devices and Laser Scanning and Recording Systems.

[27] Brown D.C. (1971). Close-Range Camera Calibration. Photogramm. Eng..

[28] Boufares O., Aloui N., Cherif A. (2016). Adaptive Threshold for Background Subtraction in Moving Object Detection using Stationary Wavelet Transforms 2D. Int. J. Adv. Comput. Sci. Appl..

[29] Luhmann T. (2023). Nahbereichsphotogrammetrie.

[30] Kuhn H.W. (1955). The Hungarian method for the assignment problem. Nav. Res. Logist. Q..

[31] He K., Gkioxari G., Dollár P., Girshick R.B. (2017). Mask R-CNN. arXiv.

[32] Davoudkhani M., Mulsow C., Maas H.G. (2025). Single camera 6-dof object tracking using spatial resection based techniques. Int. Arch. Photogramm. Remote Sens. Spat. Inf. Sci..

[33] Shorten C., Khoshgoftaar T.M. (2019). A survey on Image Data Augmentation for Deep Learning. J. Big Data.

[34] Chiu M.C., Chen T.M. (2021). Applying Data Augmentation and Mask R-CNN-Based Instance Segmentation Method for Mixed-Type Wafer Maps Defect Patterns Classification. IEEE Trans. Semicond. Manuf..

[35] Duran Vergara L., Leicht L., Beckmann B., Maas H.G. (2025). Longitudinal wave propagation determination in concrete specimen under impact loading by ultrahigh-speed camera image sequence and strain gauge data analysis. Meas. Sci. Technol..

